# Walking the fine line between OSA and aging

**DOI:** 10.1007/s11325-025-03343-x

**Published:** 2025-05-23

**Authors:** Caterina Antonaglia, Antonio Fabozzi, Alessia Steffanina, Riccardo Ture, Paolo Palange, Marco Confalonieri

**Affiliations:** 1 Pulmonology Unit, Department of Medical Surgical and Health Sciences, Hospital of Cattinara, University of Trieste, 34149 Trieste, Italy; 2 Pulmonology Unit, Department of Public Health and Infectious Diseases, Pulmonary Division, Policlinico Umberto I Hospital, Sapienza University of Rome, Rome, Italy

**Keywords:** Obstructive sleep apnea, Sleep apnea syndrome, Elderly, Aging, Phenotype, Comorbidity

## Abstract

Obstructive Sleep Apnea (OSA) is the most common sleep- related breathing disorder. In recent years, evidence have shown that patients with OSA may have this disorder for different reasons, with different symptoms and comorbidities. Therefore, treatment should be individualized [1]. This has led to a growing interest in the characterization of the disease into phenotypes. OSA in elderly patients is often a challenge for clinician in terms of diagnosis, as symptoms may be masked, but also for treatment in terms of efficacy and adherence. However, aging is a pathophysiological factor that predisposes to obstructive sleep apnea, and the two conditions share symptoms and comorbidities to such an extent that it becomes very difficult to establish a casual link between the two. We summarize the recent evidence in OSA elderly patients, particularly in terms of pathophysiology, symptoms and main comorbidities.

## Introduction

**Fig. 1:**
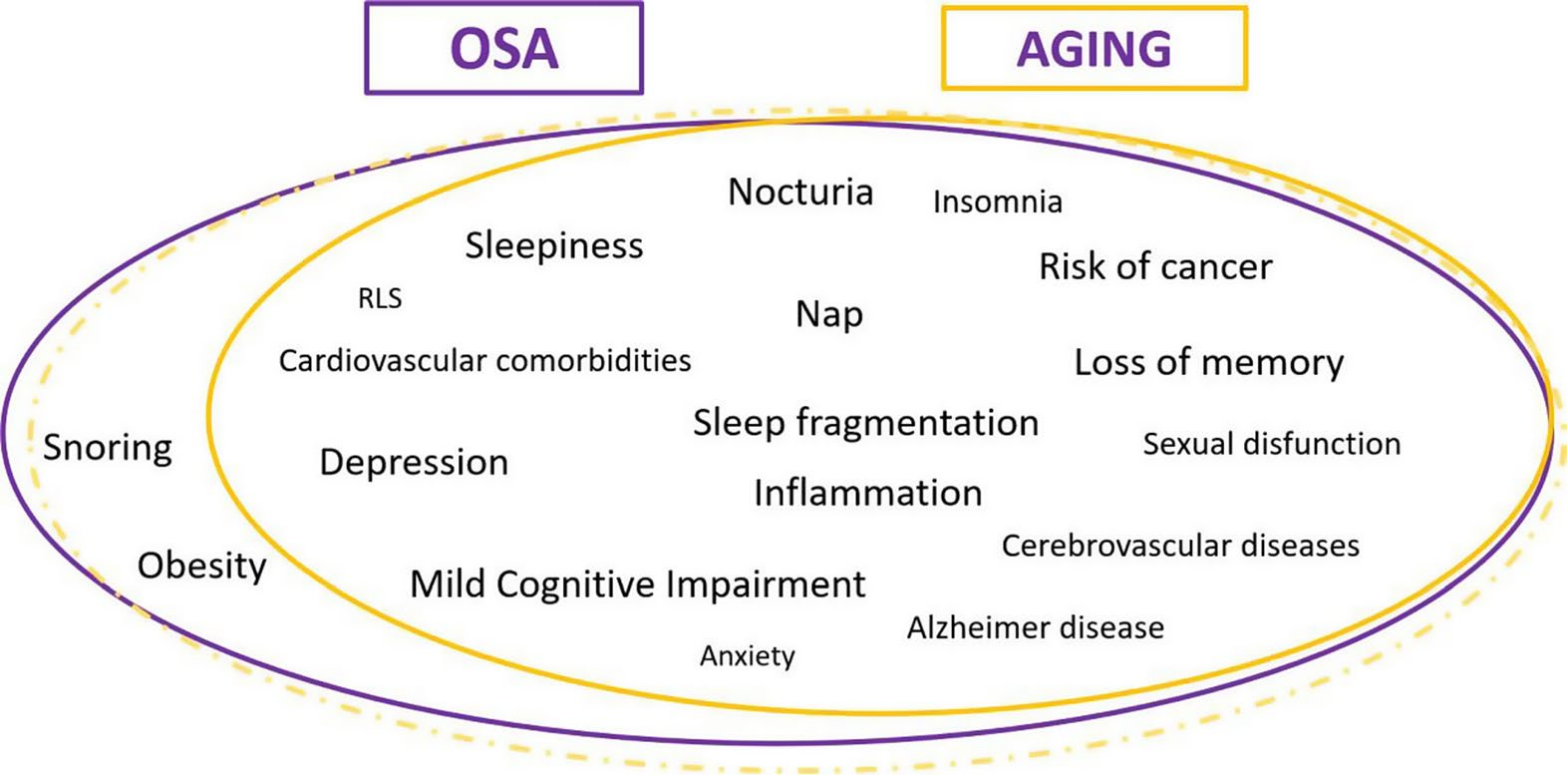
Symptoms and comorbidities in common between OSA and Aging

Wisconsin Sleep Cohort has demonstrated that male sex is no longer an important risk factor for OSA after the age of about 50 years old and this evidence has been confirmed from other studies [2]. This difference can be explained at least in part by the increased prevalence of OSA in women after entering menopause [3]. As a consequence, some investigators have reported in scientific studies that the male-to-female ratio for older people is 1:1. A recent large study demonstrated that 50% of older men had a respiratory disturbance index > 13/h, with aging being an independent risk factor in multivariate logistic regression analyses [4]. The prevalence of OSA in elderly is very difficult to define. Considering the AASM 2023 criteria for hypopnea [5], prevalence of OSA ranged around 75% for 65 years old people with AHI > 5 [6]. The reason of this so high prevalence is that OSA can be also defined by an AHI more than 5 events/h if associated with the presence of disability-related symptomatology or with the presence of comorbidity [5]. The issue concerning the prevalence of OSA in the elderly is that the classically associated symptoms of the disorder (daytime sleepiness, fatigue, cognitive dysfunction) and the comorbidities (hypertension, diabetes, cerebrovascular and cardiovascular diseases) increase in prevalence with aging. Such high prevalence of the disease raises the question whether the increased collapse of the respiratory upper tract with aging may be part of a physiological process in elderly people. In other words, is an AHI greater than 5 the right cut off in elderly? Specific symptoms or comorbidities in elderly may be secondary to another disorder, e.g. nocturia? Finally, how many patients over-65 years old do not have the classical comorbidities such as hypertension, ischemic heart disease, atrial fibrillation, cognitive impairment, diabetes mellitus, etc.? OSA-associated intermittent hypoxia may promote the induction of some hallmarks of aging like for example genomic instability, telomere shortening, epigenetic alterations, loss of proteostasis, deregulated nutrient sensing, mitochondrial dysfunction, cellular senescence, stem cell exhaustion, and altered intercellular communication [7]. In OSA also it is very difficult to distinguish when the cause of these symptoms and comorbidities is the presence of a sleep disorder breathing or aging (Fig. 1).

Nevertheless, it is important remember that aging in itself is not a disease and elderly patients are very different. This is our challenge: not to consider the symptoms of our patients always secondary to aging.

### Objectives

This narrative review aims to investigate the complex interaction between OSA and aging. The topics covered are epidemiology, pathophysiological mechanisms, symptomatology, comorbidities and therapeutic management challenges. The aim is to outline the intersecting features between aging and OSA-related events, which frequently complicate the diagnostic and therapeutic management of OSA elderly patients. This review also aims to highlight the relevance of identifying specific phenotypes in OSA elderly patients and the need for a more personalised approach in this population.

### Methods

The bibliography was screened through a focused, non-systematic search of the PubMed and Scopus databases, using the following combination of terms: “obstructive sleep apnea and aging”, “obstructive sleep apnea and elderly”. Articles were selected accordingly to their relevance and contribution to the comprehension of OSA in the elderly population. Recent reviews, guidelines and original studies received priority.

### Sleep in elderly

The evidence that melatonin levels diminish with age and reach levels similar to daytime concentrations, may contribute to the increased prevalence of sleep-related disorders with aging. Aging negatively affects the total sleep time, reduces deep sleep with increase of percentage of light sleep and little variation in percentage of REM sleep. There is also a reduction of sleep efficiency as well as an increased number of arousal. These factors are influenced by endogenous changes but also by lifestyle changes and some comorbidities more frequent in aging: insomnia, neurocognitive disorders o decline, mood disease [8]. Many scientific works have a discussion about the differences in sleep with aging and how much sleep fragmentation could result from overtime spent in bed, possibility to take nap and also how much sleep restriction could be help to improve sleep in older patients. Sleep fragmentation and reduced sleep quality in elderly are very common related to ageing itself, different lifestyle (like unscheduled activity), but also to comorbidities. Some common diseases like insomnia, depression, anxiety, chronic pain for arthritis etc… can influencing sleep quality, but also primary sleep disorders are more frequent in elderly population. Rest leg syndrome (RLS), Periodic limb movements (PLMs), REM sleep disorder or circadian rhythm disorders are the more frequent no respiratory sleep disturbances that could cause reduced sleep quality in elderly patients. OSA is very prevalent in elderly and also when the AHI is low it could be related to a significant sleep fragmentation or excessive sleepiness, as well as in younger patients. Sleep fragmentation itself is related to brain changes and impairment of cognitive function. For all these reasons, asking elderly patient about sleep quality or investigating the presence of one of the above-mentioned disorders should be part of our daily practice.

### Pathophysiology of sleep disorder breathing in elderly

People have OSA for different reasons: in 100% of patients there are one or more anatomical factors with different contribution of one or more functional factors between low arousal threshold, high loop gain or alteration in the upper airway responsiveness [1]. Two principal studies evaluated the role of functional factors in elderly OSA patients with in both a more upper airway collapsibility [9, 10]. Due in part to overall improvements in health, the population of elderly individuals is increasing rapidly. Similarly, Obstructive Sleep Apnea (OSA) is both gaining increased recognition and is also increasing due to the worldwide obesity epidemic. The overlap of OSA and aging is large, but there is strong plausibility for causation in both directions: OSA is associated with pathological processes that may accelerate aging and aging related processes; aging may cause physical and neurological changes that predispose to obstructive (and central) apneas. In addition, the common symptoms (e.g. excessive daytime sleepiness, defects in memory and cognition), possible physiological consequences of OSA (e.g. accelerated cardiovascular and cerebrovascular atherosclerosis), and changes in metabolic and inflammatory markers overlap with the symptoms and associated conditions seen in aging. There is also the possibility of synergy in the effects of these symptoms and conditions on quality of life, as well as a need to separate treatable consequences of OSA from age-related complaints. Taken together, the above make it essential to review the interaction of OSA and aging, both proven and suspected. The present review examines some aspects of what is known and points to the need for further investigation of the relationships between aging and OSA, given the large number of potentially affected subjects [10]. Martin et al. measured upper airway caliber using acoustic reflection and found that all upper airway dimensions, except for the oropharyngeal junction, decreased modestly with age [11]. Elderly people have more fat pad in the upper airway not BMI related, with a more long upper airway (especially in older women), long face and an increased pharyngeal resistance [12]. From the age of 60 onwards, obesity is no longer a statistically significant risk factor for sleep-disordered breathing [12]. In non obese men, aging is related to the overnight fluid displacement from the legs, related to prolonged sitting, to the neck and it may play a previously unrecognized role in the pathogenesis of obstructive sleep apnea that is independently from body weight [13]. Breathing during sleep in elderly people also without OSA result in airflow limitation [ 14]. In 2001, Browne et al. demonstrated that the airflow of elderly people without OSA measuring the airflow with a pneumotachograph and the resistive pressure with an esophageal catheter for several breaths presented a physiological airflow limitation during sleep [15]. Functionally, the response of the genioglossus muscle to negative pressure applied during wakefulness and sleep is reduced with aging, especially in men. The increased upper airway collapsibility combined with the reduction of upper airway reflexes and responsiveness seems to be the principal pathophysiological factor of OSA in elderly people [6]. Despite these changes, the central control of breathing is relatively stable in older people, although arousal frequency increases with age. Arousal from sleep leads to hyperventilation and relative hypocapnia, which may promote respiratory instability and periodic breathing during the subsequent period of sleep onset. This mechanism could explain the occurrence of central apnea at sleep onset in some elderly patients. Edwards et al. demonstrated a marginal role of high loop gain or of low arousal threshold in the development of OSA in elderly people respect to younger ones, while increased airway anatomical collapsibility plays a predominant pathogenic role [10]. A possible explanation is that, over time, untreated OSA leads to a more collapsibility of upper airway due to aging-related anatomical alterations, and to a progressive inflammation from repetitive open and closure events of the upper airway resulting in a prevalent anatomical phenotype of OSA. Nevertheless, with aging there are also changes in the functional factors. Chemoreceptors become less sensible, possibly leading to a more stable ventilatory pattern in elderly people compared to younger. It is important to underline that loop gain in the elderly could be modified in terms of mixing gain by cardiovascular conditions. In particular, a concomitant heart failure or more frequently atrial fibrillation can increase time of circulation and represented risk factor for Cheyne Stokes breathing pattern or periodic central apneas. In fact, while controller gain is generally reduced with aging, plant (lung interstitial edema) and mixing gain could be altered by the more frequent cardiovascular comorbidities. As concern low arousal threshold (lowAT), although aging is typically related to sleep fragmentation and poor quality of sleep, lowAT it does not represent a major risk factor for OSA in elderly. An explanation could be that, in elderly, the negative endoesophageal pressure required to reopen the airway with reflex mechanisms (named threshold of mechanical recruitment) is less negative than the negative pressure necessary to trigger cortical arousal. This means that older OSA patients may reopen the upper airway without arousal compared to younger patients [16, 17]. Poor responsiveness of the upper airway is an important risk factor in aging for OSA onset. For example, postmenopausal women will experience an increasing risk for OSA due to the reduced levels of sexual hormones with their trophic neuromuscular effects on upper airway muscles [18].

### Symptoms and diagnostic tools

In older patients, aging and OSA have many symptoms in common. The shared symptoms makes diagnosing OSA very difficult and screening tools less effective than in younger patients. In some cases, patients or clinicians underestimate the disorder and attribute symptoms such as memory loss, difficulty concentrating, sleepiness, etc. to ageing, on the other hand, our elderly patients very often suffer from one or more sleep disorders involving the respiratory system or not. Suspicion of sleep-disordered breathing difficulties is a challenge because screening tools are not specific for ageing patients. The gold standard for the diagnosis of OSA, also in the elderly, is the complete Polysomnography (PSG). In older patients, the frequent presence of comorbidities like insomnia, Restless Legs Syndrome (RLS), Periodic Limb Movements (PLMs), REM sleep behavior disorders etc. makes it even more important the use of PSG, despite the fact that home sleep test (HSAT) remains the most frequently performed test in routine practice [5]. In the Wisconsin Sleep Cohort, the prevalence of PLMs with an index greater than 15 was 28.8% in patients with a mean age of 56.1 years, but reached 43.4% at a mean age of 65.0 years, highlighting a significant increase in prevalence with age [19]. Older adults with OSA are also more likely to experience PLMs. The aging process itself is associated with changes in the central nervous system that could make PLMs more likely. Additionally, sleep fragmentation from OSA in older individuals may exacerbate the occurrence of PLMs. In older patients, we could find coexisting central sleep apnoea related to heart failure, atrial fibrillation, insomnia, drugs such as hypnotics, renal failure, cerebrovascular disease, which are more common than in younger patients. Some studies have shown that the overall prevalence of CSA in a community population was 0.9%, versus CSA in those aged > 65 years of 1.8% (female) and 2.7% (male) [20]. In the case of hypopnea, differentiating between central and obstructive events is not always easy, while the presence of central sleep apnoea completely changes our diagnostic and therapeutic approach. The coexisting and prevalent insomnia in the elderly population is another important aspect associated with the difficulty to recognize the presence of OSA, or in the case of the two disorders coexistence of Co- Morbid Insomnia and Sleep Apnea (COMISA) [21]. COMISA or insomnia-like symptoms can increase the risk of major adverse cardiovascular events in patients with OSA, and identifying these patients is important for clinical personalized treatment [22]. All of these comorbidities could interfere with the diagnosis and treatment of our patients. In clinical practice, a full polysomnographic study is not feasible in terms of cost and number of patients, and it also interferes with sleep. For these reasons, scoring sleep studies in elderly patients, after a full clinical evaluation, requires experience in this field. The value of HSAT is considered appropriate for patients with a high pre-test probability, usually estimated with validated questionnaires. Berlin and STOPBANG questionnaire have a good predictivity in term of specificity and sensibility respectively [23]. In general in elderly patients the symptoms and natural history may be different compared to the middle-aged patients: elderly people with OSA are usually less symptomatic than young OSA patients. Elderly OSA patients do not complain of typical snoring, apnea or choking during sleep, while nocturia, sexual dysfunction and memory loss are usually more common. However, symptoms like fatigue, sleepiness, cognitive impairment, loss of memory could be attributed to aging. Anxiety and depression, which are more common in older patients, are another reason to pay attention to our patients’ medical history. Therefore, the clinical suspicion of OSA in the elderly can be very challenging. Differentiating OSA symptoms from age-related changes requires a thorough clinical evaluation, including history, physical examination and sleep studies, especially in the presence of common comorbidities. Nevertheless, sleepiness is the most difficult symptom to assess in aging people. Sleepiness in elderly people may be the consequence of the aging-related physiological sleep fragmentation combined to the reduced daytime activity and the common habit of daytime napping. Consequently, elderly people often do not refer excessive daytime sleepiness (EDS). Therefore, the evaluation of EDS with Epworth Sleepiness Scale (ESS) has shown a limited predictive value in this population [19]. As previously demonstrated, subjective sleepiness is reduced with aging: elderly OSA patients report less EDS than younger OSA patients but more EDS respect to elderly control without OSA [24]. In elderly people some studies reported also a reduction in objective sleepiness with aging, particularly comparing people over 60 years old in respect to younger adults [25]. However, stronger evidence may be missed. Moreover, a cut-off point of 10 on the ESS could be inappropriate, due to a possible age-related increase in EDS related to comorbidities, inactivity, obesity, psychotropic medication, Alzheimer, depression, mild cognitive impairment etc. Previous studies showed that subjective sleepiness is also reduced in the elderly. In fact, older OSA patients, report less sleepiness compared to younger OSA patients at ESS. A strong and important finding is that the presence of EDS secondary to sleep disorder breathing with an AHI more than 20 events per hour was associated with an increased mortality risk in older adults, even when adjusting for other significant risk factors, compared to EDS related to other disorders [26]. To better define the phenotype of OSA in the elderly, several studies proposed a cluster analysis of OSA patients. Ida et al. tried to divide the OSA Japanese population in different clusters [27]. The elderly cluster with a mean age of 70 years old showed: lower obesity rates, fewer symptoms, a lower ESS but a high burden of comorbidities, including hypertension and cardiovascular diseases. This cluster also showed specific PSG findings, including a shorter total sleep time, a lower percentage of total sleep time and a reduced percentage of N3 and REM sleep. The mean AHI of this cluster indicated a high prevalence of severe OSA [28]. Other studies confirmed that elderly OSA patients usually has fewer symptoms, more comorbidities and a lower ESS score compared to younger patients.^29^ Older patients report sleepiness in different way and in particular some items of the ESS may not be appropriate for the lifestyle of elderly people [30]. For example, ESS does not consider that afternoon naptimes may not be pathological but rather part of a normal daily routine.

### Comorbidities

OSA elderly patients may not suffer the same health consequences as their younger counterparts. Cognitive impairment is severe, cardiac arrhythmias and heart failure are common. Nevertheless, also metabolic and cerebrovascular diseases are descripted as associated with OSA in elderly patients.

#### Cognitive impairment

A study published in Jama in 2011 about 300 elderly women with OSA showed that they have an increased risk of developing Mild Cognitive Impairment (MCI). The association was greater with oxygen desaturation index (ODI) and the time spent below SatO2 < 90% (T < 90%) respect to sleep fragmentation, supporting the hypothesis that the elderly brain is physiologically less sensitive to sleep fragmentation [31]. OSA is commonly associated with neurocognitive impairments that have not been consistently related to specific brain structure abnormalities. Nevertheless, some studies try to evaluate Magnetic Resonance Imaging (MRI) alteration in OSA patients. For example Canessa et al [31]. performed a particular MRI scans in the examined patients to assess volumetric changes in grey matter in specific brain areas (hippocampus and frontal) and revealed a focal volumetric reduction in OSA compared to controls. These brain changes were improved with Continuous Positive Airway Pressure (CPAP) therapy and the improvement correlated with specific neurocognitive tests. Numerous studies showed that patients with OSA develop MCI and Alzheimer Disease (AD) at an earlier age than their counterparts and CPAP treatment slow the progression of MCI [32]. The most recent study on the subject is that of Marchi et al [33]. This study uses data from the Hypnolaus to analyze the association between OSA in the elderly (358 patients), aged approximately 71 years, and to assess cognitive alterations at 5 years. The most significant polysomnographic indexes like mean saturation (when less than 92.5%) and tT < 90% (4.5%) where most associated with cognitive decline as measured by the mini mental state examination [33]. The greater role of desaturation levels respect to intermittent hypoxia suggests that the brain may be more vulnerable to hypoxia than to phasic desaturations. On the other hand, indices such as AHI and ODI and hypoxic burden were associated with decline only in the subcategory of older and male patients with ApoE4 alterations (i.e. patients with risk factors). Sleep fragmentation does not appear to play a role in determining cognitive impairment. Previous studies have shown that women compared to men have cognitive decline only if they have OSA but in this study women were only post-menopausal. Studies show that OSA can initiate the process of cognitive impairment earlier in both ageing and AD. Different randomized clinical trials (RCT) provide an insight into the causal associations between OSA and AD and are more compelling. All RCTs were conducted in older adults and showed that CPAP treatment not only improved sleep parameters (e.g., SWS, EDS) in AD patients with OSA, but it also increased cognitive function. These findings provide evidence that AD patients (particularly mild to moderate) with OSA may benefit from CPAP treatment [34]. The hippocampus is one of the main and most consistently brain region that correlates with mild cognitive impairment. We do not have drugs to treat cognitive impairment in Alzheimer so when sleep disorders breathing coexist with cognitive decline in a patient with a higher risk of AD, the use of CPAP is the best strategy to prevent the progressive cognitive impairment [31]. Some evidences suggest that disturbances in general Intellectual function and executive function show strongest correlations with measures of hypoxemia. Not unexpectedly, alterations in vigilance, alertness, and, to some extent in memory, seem to correlate more with measures of sleep disruption.

#### Cerebrovascular disease

OSA has been identified as one of the major risk factor for cerebrovascular disease and chronic intermittent hypoxia (CIH) is considered the principal mediator of this increased risk [35, 36]. In fact, the cerebrovascular blood flow (CBF) response to chronic intermittent hypoxia in severe OSA is negatively correlated with AHI and time with SaO2 < 90% during sleep. So, in severe OSA, there is a lower hypoxic cerebrovascular reactivity compared to health subjects [37, 38]. OSA developed in older age seems to have a lower susceptibility to the negative effects of OSA-induced chronic intermittent hypoxia compared to younger patients [39]. In fact, with aging, the arousal threshold (AT) is reduced and sleep fragmentation is higher [40]. This results in a earlier breathing response to an apnea or hypopnea event, so older patients develops less respiratory events and less nocturnal hypoxaemia compared to the younger patients. Another mediator of cerebrovasculature damages in older patients with OSA in impaired autoregulation of CBF: this is the mechanism which by the CBF maintains a relatively constant blood flow when perfusion pressure changes [41]. This mechanism is impaired in severe OSA, so the cerebral blood flow changes in response to modifications of systemic blood pressure (hypertension or hypotension) and the risk of cerebral ischemia increases [42]. In a recent inpatient sample analysis of 1,141,120 G-OSA (geriatric OSA) patients, 9,9% of them presented major adverse cardiac and cerebrovascular events (MACCE) during their hospitalization [43]. After multivariable regression analysis, the major significant clinical predictors of MACCE were pulmonary circulation disease, coagulopathy, peripheral vascular disease, prior sudden cardiac arrest, prior myocardial infarction, fluid and electrolyte imbalances, male sex, hyperlipidemia, renal failure, T2DM, metastatic cancer and prior stroke or TIA [44]. In a prospective study of OSA patients admitted for newly diagnosed MI at Mayo Clinic (Rochester, Minnesota), an AHI of ≥ 15 events per hour, a minimum arterial oxygen saturation (MinSaO2) of ≤ 85% and an Epworth Sleepiness Scale scores of ≥ 11 have emerged as independent MACCE risk factors over a 3–5-year follow-up [45]. In a prospective cohort study of elderly patients with OSA, the risk of mortality secondary to ischemic stroke increased for untreated patients with severe OSA while the treatment with CPAP decreased the risk in this group to levels similar to patients without OSA [46]. In a post hoc analysis of a prospective observation study which examines the risk of ischemic stroke in the elderly population with OSA and the effect of CPAP-therapy, the adjusted HR ratio for the incidence of stroke were 3.42, 1.02, and 1.76 for the untreated severe OSA group, CPAP-treated group, and untreated mild-moderate OSA group, respectively, when compared with the reference group [47]. These findings in these studies specifically performed in the elderly severe OSA suggests that there is an increased risk of ischemic stroke in this population and CPAP may reduce this risk.

#### Cardiovascular diseases

In literature studies about cardiovascular comorbidities and OSA in elderly have the intrinsic limitations of the observational studies. So in 2012, a particularly interesting retrospective study [45] was conducted on 939 patients aged at least 65 years and were followed up for a mean of 69 months after being referred to a sleep specialist with suspicion of OSA. The severe cases that refused CPAP treatment or showed bad compliance presented excessive mortality from cardiovascular and cerebrocardiovascular apart from coronary disease. In 2019 other authors decide to da a post hoc analysis [46] that confirm the previous results. The incidence of stroke, but not coronary heart disease, is increased in elderly patients with untreated severe OSA. Adequate CPAP treatment may reduce this risk. For these results were postulated more hypothesis. The reduced cardiovascular risk could be attributable to reduction of post- event sympathetic activation in the elderly or protective effect of intermittent hypoxia that favors ischemic preconditioning at the cardiac level [47]. In the last few years literature explains the failure of CPAP to reduce the cardiovascular and metabolic risk, comorbidities or mortality in OSA patients because all studies in the previous years consider the AHI as the target therapy to considered. Recent literature considers other index like hypoxic burden, mean of desaturation value, time under 90% oxygen saturation to relate risk of cardiovascular disease [48]. In elderly population we do not know which polysomnographic index between AHI, ODI, T < 90%, hypoxic burden etc. is better related to cardiovascular comorbidities. When critically analysing the studies that failed to demonstrate the efficacy of CPAP on coronary risk, it is important to note that almost all of the studies were confounded by poor adherence, were observational and used composite outcomes. on the other hand, elderly patients have an increased coronary risk that may be related to other comorbidities and may not be reduced by treatment for OSA. Some authors also speculate that the efficacy of CPAP in reducing coronary risk may apply to certain phenotypes, such as patients with somnolence or insomnia, who are usually excluded from randomised clinical trials.

#### Metabolic syndrome

Metabolic syndrome (MS) is one of the comorbidities of greatest scientific interest associated with OSA, as both are associated with high cardiovascular risk. The estimated prevalence of OSA in MS patients and the prevalence of MS in the OSA population was found to be 60% in both cases, with OSA patients having a 6–9 times higher risk of developing MS compared to the general population [49]. Chronic intermittent hypoxia (CIH) secondary to OSA is the most important trigger for the metabolic dysfunction typical of MS. Chronic intermittent hypoxia in fact stimulates hyper-activation of the sympathetic nervous system, increasing circulating inflammatory products and the production of reactive oxygen species [50]. A cohort study selectively performed on a group of healthy elderly people, the SYNAPSE cohort study, aimed to investigate the association between MS and OSA [51]. In this study, 12.5% of OSA group had SM while only 5% in the non-OSA group, but the SM prevalence increased in the severe OSA-group (46%). OSA elderly patients with SM presented higher BMI, higher diastolic blood pressure, higher AHI and OHI, higher insulin-resistance, higher glycemia and triglycerides. OSA is a risk factor for the development of type 2 diabetes mellitus (T2D). In fact, OSA and its severity is strongly correlated with the incidence of T2D, independently by body mass index (BMI) [52]. The etiopathogenetic hypothesis of OSA-associated T2D is supported by the beneficial effects of continuous positive airway pressure (CPAP) on T2D: in fact, the use of CPAP in diabetic patients has been shown to reduce postprandial glycaemia and glycated hemoglobin [53]. The impact of CPAP therapy in improving metabolic syndrome parameters is still controversial. The TREATOSA-MS trial, a randomized placebo-controlled trial that studied the effect of CPAP therapy in OSA + MS patients, showed that the MS reversibility rate was significantly higher in the CPAP-treated group compared to placebo-group, but most patients retained MS diagnosis after 6 months of CPAP therapy [54]. These findings show that CPAP therapy does not modify the main metabolic parameters of MS. It may be interesting to study the effect of CPAP, in addition to weight-loss interventions, on the regression of MS in patients with OSA.

### OSA treatment in elderly patients

To date, CPAP remains the first-line treatment for moderate to severe OSA in elderly patients [ 55]. A study published in the European Respiratory Journal (ERJ) in 2015 demonstrated that CPAP improved quality of life, symptom control, anxiety, depression and neurocognitive aspects in patients over 70 years old with severe OSA [56]. Similarly, CPAP treatment resulted in a significant improvement in daytime sleepiness, some sleep-related symptoms and quality of life in elderly patients with moderate OSA [57]. However, outcomes and adherence for CPAP therapy in very elderly patients is unknow [58] Evidence from the four principal clinical trials involving elderly people with OSA showed an improvement in ESS (mean reduction of -2.62 points), quality of life, symptoms, anxiety, depression and cognitive impairment. Nevertheless, the quality of evidence is still low [59]. The more questionable aspects of OSA in elderly remain the adherence to CPAP. In fact, CPAP adherence shows a linear and progressive decline in OSA patients aged ⩾65 years, peaking beyond 75 years. This reduced adherence occurs even though CPAP indication in very elderly patients is probably more selective than in younger cases. Interestingly, recent studies showed a comparable CPAP adherence between older people (but without a very elderly subgroup) and the younger subgroup [60, 61]. These controversies in literature depends on the patients’ average age, the follow up time, the nature of the study (observational or clinical trials) and the setting (community or nursing home in case of older people). Another interesting finding is that older patients with OSA-related symptoms (EDS or mood disturbance) usually show a better CPAP adherence [62]. In 2016, the Geriatric Sleep Medicine Task Force confirmed that CPAP remains the best treatment in terms of efficacy, especially for the reduction of cognitive impairment and of cerebrovascular risk [55]. The authors recommended a less aggressive approach with lower pressure of CPAP. In addition to CPAP treatment, sleep hygiene rules and weight loss are general recommendations for patients with all type of sleep disorders.

Nonetheless, alternative therapies should be considered in mild-to-moderate OSA or in patients who are CPAP-intolerant. These include:

- Mandibular Advancement Devices (MAD), particularly in non-edentulous patients without severe anatomical abnormalities 63; MAD are an alternative treatment option for highly selected patients. The patients should be evaluated by a qualified dentist to determine candidacy for an oral appliance based on the health of the dentition and existing dental work, the relationship of the maxilla to the mandible, the range of motion of the mandible, and a history of temporomandibular disorders.
- Positional therapy in another alternative therapeutic approach in OSA patients with Positional Obstructive Sleep Apnea (POSA). The first problem is that in literature there doesn’t exist a consensus in the POSA polysomnographic criteria definition. Nevertheless the definition with the higher rate of success of positional therapy is an AHI supine: non supine ratio of 4:1 with an AHI non supine less then 10 [64]. Nevertheless positional treatment in OSA patients has a very low adherence rate over time and it could be difficult in elderly patients when articular or muscular disturbance coexist [65].;
- Auto-CPAP in elderly patients should be considered in selected cases, such as positional OSA. In fact, OSA in aging presents a major association of POSA compared to younger patients [66] and in these cases an auto CPAP with low range of pressure may be an option [55]. Auto-CPAP should be avoided in elderly people with high sleep fragmentation, especially with large range of pressure [67].
- In literature the best alternative approach in patients with moderate and severe OSA is the use of BiPAP in which a different pressure is provided during inspiration versus expiration give a higher percentage of adherence [68].
- In older obese patients, bariatric surgery is not a therapeutic option. Newer drugs such as tirzepatide, a long-acting glucose-dependent insulinotropic polypeptide receptor and glucagon-like peptide-1 receptor agonist, have shown an improved safety profile, although specific studies in older populations have not yet been conducted [69].
- Hypoglossal nerve stimulation in older adults with OSA is an option for patients with predominantly poor neuromuscular responsiveness. However, studies in unselected patients have shown an increase in insomnia, sleep-related impairment and symptoms of depression that is much higher than that observed in the general population. These observations make this treatment option less applicable in elderly patients with physiological sleep disorders [70].
- Large, controlled trials of drug therapy for sleep-disordered breathing in older people are still lacking. Two small randomized trials are investigating the role of donezepil in the treatment of OSA and acetazolamide in the treatment of central sleep apnoea. Both drugs have been shown to be effective in treating sleep-disordered breathing in an elderly population, with mild (acetazolamide) or moderate (donezepil) effects [55].
- Significant improvements in sleep parameters and OSA severity after the combined exercise program may indicate an effective non-pharmacological intervention for treating these conditions in older adults [71].

Nevertheless, in the last few years the concept of target therapy suggest that in patients with a prevalent anatomical collapsibility trait (which is frequent in elderly), CPAP therapy represents the gold standard [72] However, therapeutic settings may need to be adjusted, for example using lower pressure targets [16], as elderly patients may benefit from gentler titration and more stable pressure delivery. Choosing the right interface is critical, particularly in elderly, for facial changes due to aging or edentulism. Nasal masks are the best first choice for PAP therapy in elderly patients and their use is even more important in edentulous patients with a longer face related to aging. Choosing the wrong mask may be another reason of reduced adherence in OSA elderly patients [73].

## Discussion

**Fig. 2:**
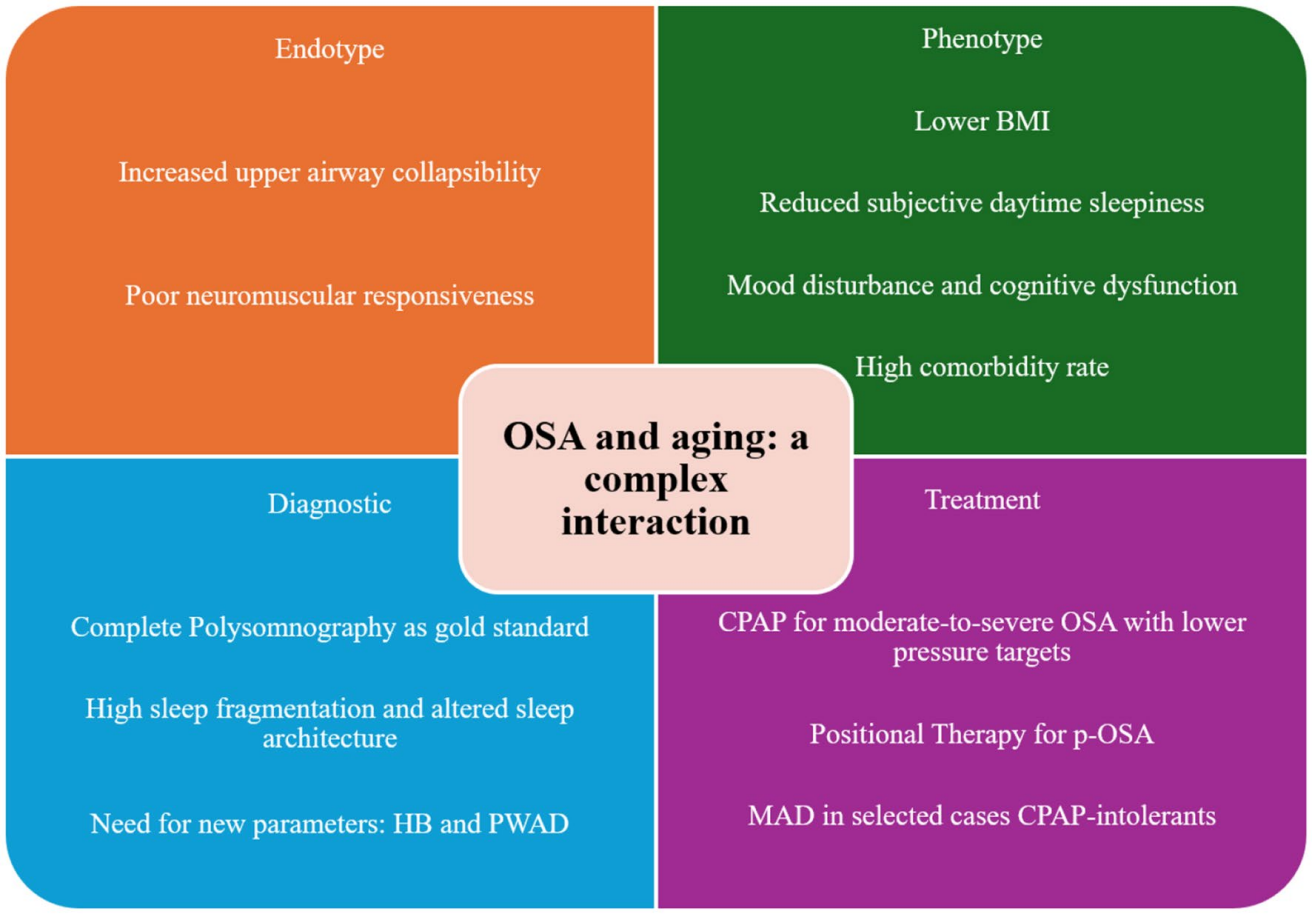
A graphical summary of findings from this narrative review. BMI, body mass index; HB, hypoxic burden; PWAD, pulse wave amplitude drops; CPAP, continuous positive airway pressure; p-OSA, positional obstructive sleep apnea; MAD, mandibular advancement device

The prevalence of OSA increases in the elderly, especially in postmenopausal women [74]. However, the clinical presentation is different in elderly OSA compared to younger ones. Classic OSA-symptoms, such as snoring and witnessed apneas during sleep are often absent, while non-specific symptoms, such as fatigue, memory disturbances or nocturia, are more frequent in OSA older patients. However, these non-specific symptoms may be attributed to aging and comorbidities. For this reason, elderly patients may be misdiagnosed or experience a more difficult OSA diagnostic process. From a pathophysiological point of view, the most frequent endotype in elderly OSA is the increased upper airway collapsibility associated with reduced neuromuscular reflexes. The low arousal threshold and high loop gain appear to be less relevant in elderly OSA, thus highlighting the role of aging as a cofactor in the pathophysiology of OSA. Complete Polysomnography remains the gold standard for the diagnosis of OSA even in the elderly, especially in suspected comorbidities such as insomnia or REM sleep disorder. Even severity indices such as the AHI, ODI or T90 do not fully consider the burden of OSA in the elderly. Therefore, the more recent polysomnographic indices for prognostic information in elderly OSA, such as the Hypoxic Burden [75] and the Pulse Wave Amplitude Drops [76], may be useful for a complete evaluation of OSA older patients. Furthermore, conventional instruments such as ESS have limited predictive value in the elderly due to different lifestyles and reduced perception of both objective and subjective sleepiness. There is a necessity to develop new screening instruments for OSA specifically for the elderly, such as equations or scores based on morphological and anthropometric parameters [77, 78]. Comorbidities impact on the clinical outcomes of OSA in the elderly. This impact should be reduced by a good CPAP adherence. In fact, CPAP therapy improves structural brain changes and cognitive function in MCI and AD and reduces the risk of cerebrovascular and cardiovascular events by lowering intermittent hypoxia and autonomic dysfunction. CPAP is confirmed as the first-line treatment in moderate-to-severe OSA in elderly, improving EDS, mood, cognitive function and quality of life. However, alternative therapies should be considered in selected cases. A graphical summary is present in Fig. 2.

## Conclusion

OSA in the elderly represents a specific and evolving challenge, often under-diagnosed due to non-specifical symptoms and comorbidities. While aging drives the pathophysiology of OSA in older patients, the diagnosis and management of these patients should be personalized following the “target therapy” concept. As the average age of population is still growing, clinicians should be aware of the distinct phenotype of OSA in the elderly and should utilize specific diagnostic tools and personalized treatments, considering the functional status, comorbidities and patient preferences.

Further studies for OSA in elderly are needed to investigate new screening tools, elaborate diagnostic thresholds and optimize treatment and management of this increasing population.
